# Host candidate gene polymorphisms and clearance of drug-resistant *Plasmodium falciparum *parasites

**DOI:** 10.1186/1475-2875-10-250

**Published:** 2011-08-25

**Authors:** Mahamadou Diakite, Eric A Achidi, Olivia Achonduh, Rachel Craik, Abdoulaye A Djimde, Marie-Solange B Evehe, Angie Green, Christina Hubbart, Muntasir Ibrahim, Anna Jeffreys, Baldip K Khan, Francis Kimani, Dominic P Kwiatkowski, Wilfred F Mbacham, Sabah Omar Jezan, Jean Bosco Ouedraogo, Kirk Rockett, Kate Rowlands, Nawal Tagelsir, Mamadou M Tekete, Issaka Zongo, Lisa C Ranford-Cartwright

**Affiliations:** 1 Malaria Research and Training Centre, Faculty of Medicine, Pharmacy and Odontostomatology, University of Bamako, Mali; 2 University of Buea, Faculty of Health Sciences, Department of Medical Laboratory Science, B.P. 63 Buea, S.W.P. Province, Cameroon; 3 Université de Yaounde I, Centre de Biotechnologie, B.P. 8094, Yaounde, Cameroon; 4 Wellcome Trust Centre for Human Genetics, University of Oxford, Oxford, UK; 5 Department of Molecular Biology, Institute of Endemic Diseases, University of Khartoum, Khartoum, Sudan; 6 International Atomic Energy Agency (IAEA), Vienna, Austria; 7 Kenya Medical Research Development, Malaria Unit, Centre for Biotechnology Research & Development, Mbagathi Road, P.O. Box 54840, Nairobi, Kenya; 8 Wellcome Trust Sanger Institute, Hinxton, Cambridgeshire, CB10 1SA, UK; 9 Direction régionale de l'Ouest, 399, Avenue de la liberté, Institut de recherche en sciences de la santé (IRSS), 01 B.P. 545 Bobo Dioulasso 01, Burkina Faso; 10 National Ministry of Health, National Health Laboratory, Department of Parasitology, P.O. Box 287, Khartoum, Sudan; 11 Institute of Infection, Immunity and Inflammation, College of Medical, Veterinary and Life Sciences, University of Glasgow, 120 University Place, Glasgow G12 8QQ, UK

## Abstract

**Background:**

Resistance to anti-malarial drugs is a widespread problem for control programmes for this devastating disease. Molecular tests are available for many anti-malarial drugs and are useful tools for the surveillance of drug resistance. However, the correlation of treatment outcome and molecular tests with particular parasite markers is not perfect, due in part to individuals who are able to clear genotypically drug-resistant parasites. This study aimed to identify molecular markers in the human genome that correlate with the clearance of malaria parasites after drug treatment, despite the drug resistance profile of the protozoan as predicted by molecular approaches.

**Methods:**

3721 samples from five African countries, which were known to contain genotypically drug resistant parasites, were analysed. These parasites were collected from patients who subsequently failed to clear their infection following drug treatment, as expected, but also from patients who successfully cleared their infections with drug-resistant parasites. 67 human polymorphisms (SNPs) on 17 chromosomes were analysed using Sequenom's mass spectrometry iPLEX gold platform, to identify regions of the human genome, which contribute to enhanced clearance of drug resistant parasites.

**Results:**

An analysis of all data from the five countries revealed significant associations between the phenotype of ability to clear drug-resistant *Plasmodium falciparum *infection and human immune response loci common to all populations. Overall, three SNPs showed a significant association with clearance of drug-resistant parasites with odds ratios of 0.76 for SNP rs2706384 (95% CI 0.71-0.92, P = 0.005), 0.66 for SNP rs1805015 (95% CI 0.45-0.97, P = 0.03), and 0.67 for SNP rs1128127 (95% CI 0.45-0.99, P = 0.05), after adjustment for possible confounding factors. The first two SNPs (rs2706384 and rs1805015) are within loci involved in pro-inflammatory (interferon-gamma) and anti-inflammatory (IL-4) cytokine responses. The third locus encodes a protein involved in the degradation of misfolded proteins within the endoplasmic reticulum, and its role, if any, in the clearance phenotype is unclear.

**Conclusions:**

The study showed significant association of three loci in the human genome with the ability of parasite to clear drug-resistant *P. falciparum *in samples taken from five countries distributed across sub-Saharan Africa. Both SNP rs2706384 and SNP1805015 have previously been reported to be associated with risk of malaria infection in African populations. The loci are involved in the Th1/Th2 balance, and the association of SNPs within these genes suggests a key role for antibody in the clearance of drug-resistant parasites. It is possible that patients able to clear drug-resistant infections have an enhanced ability to control parasite growth.

## Background

*Plasmodium falciparum *malaria remains a major cause of morbidity and mortality among children and pregnant women in sub-Saharan Africa. The most recent global figures show that malaria was responsible for over 863,000 deaths in 2008 and one fifth of the world's population is at risk [1]. 85% of cases and 89% of deaths due to malaria are found in sub-Saharan Africa [1]. Over the last decade some African countries have seen a reduction in malaria cases and deaths, probably through increased funding for disease control measures such as the use of insecticide-treated mosquito nets. However parasite resistance to anti-malarial drugs, and mosquito vector resistance to insecticides, remain a major threat to the control of malaria.

Development of acquired immunity to malaria, which is only partially protective, requires persistent, sub-clinical infection over a period of several years (reviewed in [2]). The partial protection is strain-, stage- and species-specific. This may account for the observed higher malaria infection in children than in adults, and indicates that the immune status of the host influences the severity of malaria disease and the outcome of the treatment [3].

It is known that host genetic factors play a significant role in determining an individual's susceptibility to many infectious diseases, including malaria [4-6]. Factors such as ethnic background [7], immunity [8,9], age [10], drug availability [11], co-infecting pathogens [12], socio-economical status [13], and parasite population structure [14] may impact on the outcome of infection, and the development of an effective immune response.

Advances in molecular biology have led to the discovery of genes involved in resistance to commonly used anti-malarial drugs such as chloroquine and sulphadoxine-pyrimethamine [15,16]. However the prevalence of parasites carrying the "resistant" alleles of these genes consistently exceeds *in vivo *treatment failure rates in malaria endemic settings [17], implying that some human hosts in malaria endemic-areas are able to clear genuinely drug-resistant malaria parasites. The ability to clear resistant parasites is associated with age [10,18], suggesting that host acquired immunity has a critical role in the clearance of drug-resistant *P. falciparum *infections in endemic regions. Several studies have supported the role of antiparasite immune responses in the therapeutic response to anti-malarial drugs during acute malaria ([19,20], reviewed in [3]). Host genetic factors such as sickle cell trait (HbAs), alpha-thalassaemia and haemoglobin E, as well as host pharmacogenetic differences, can also have an impact on the outcome of treatment with anti-malarial drugs [21-24]. The outcome of anti-malarial chemotherapy is, therefore, dependent on host genetic and immunological factors, as well as the level of drug resistance shown by the parasites.

In this study, known host genetic factors (other than haemoglobinopathies) that might account for individual differences in the clearance of drug-resistant parasites have been analysed in samples taken from subjects aged from 5 months old. The study included data from five African countries from both West and East Africa. The human gene variants investigated included cytokines and other immune mediators, thought to be involved in malarial pathogenesis, together with their receptors, and promoters. The overall objective of this study was to identify host immune factors that may be responsible for *in-vivo *clearance of drug-resistant *P. falciparum *by comparing allele frequencies of known SNPs in patients who clear genotypically resistant parasites with those patients who do not.

## Methods

### Study location and participant recruitment

**Figure 1:**
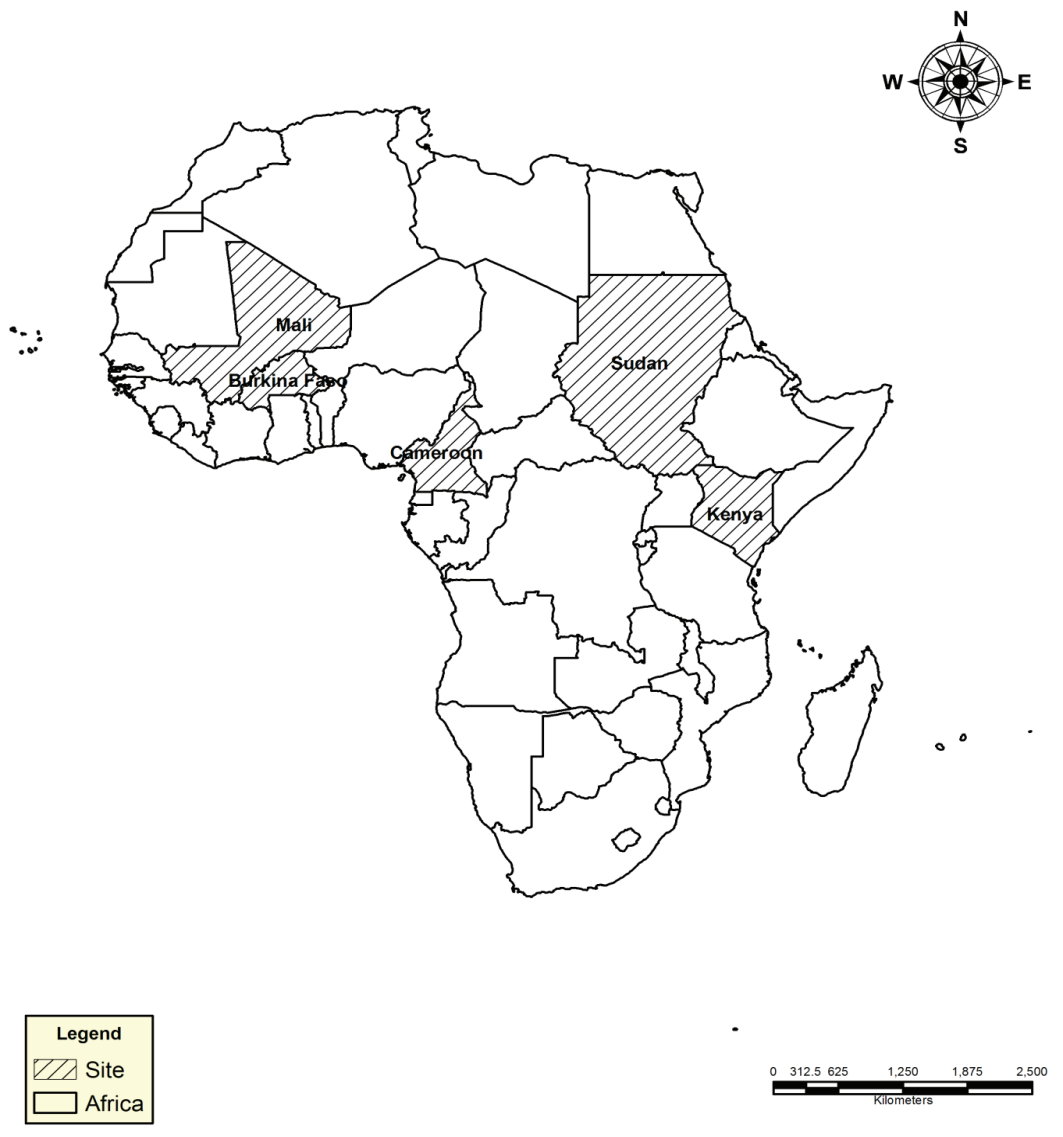
**Study sites in Africa**. Countries involved in the project are shaded.

Individuals were recruited to the study from five African countries: Burkina Faso, Cameroon, Kenya, Mali and Sudan (Figure 1). These countries were members of the International Atomic Energy's Co-ordinated Research Project E15019 on "Improved accuracy and immunological markers for prediction of efficacy of anti-malarial drugs". In all study sites, *P. falciparum *is responsible for > 95% of the clinical cases.

Individuals aged from 5 months old, with uncomplicated *P. falciparum *malaria, who were treated with antimalarial drugs including chloroquine, amodiaquine, sulphadoxine-pyrimethamine (SP) and artemisinins according to the policy within each country, were recruited to standard *in vivo *drug efficacy studies carried out in accordance with WHO protocols [25]. Details of these studies and their outcomes have been previously reported: Burkina Faso [26], Sudan [27], Cameroon [28,29], Kenya [30] and Mali [31], and a summary is provided in Table 1.

**Table 1:** Molecular Summary of *in vivo *drug efficacy trials carried out by the participant countries

Country	Drugs studied in efficacy trials *in vivo*	Follow-up period (days)	Age range of study participants
Burkina Faso	Dihydroartemisinin + piperaquine	42	6 months - 53 years
	
	Artemether + lumefantrine	28/42	6 months - 39 years
	
	Amodiaquine	28	6 months - 18 years
	
	Amodiaquine + artesunate	28	6 months - 30 years
	
	Amodiaquine + sulphadoxine-pyrimethamine	42	6 months - 55 years

Cameroon (Yaoundé)	Sulphadoxine-pyrimethamine	28	5 - 59 months
			
	Amodiaquine		
			
	Amodiaquine + sulphadoxine pyrimethamine		

Cameroon (Buea)	Artesunate + sulphadoxine-pyrimethamine	28	6 - 60 months
			
	Amodiaquine + artesunate		

Kenya	Chloroquine	14	5 months-18 years
			
	Sulphadoxine-pyrimethamine	28	
			
	Sulphadoxine pyrimethamine + Cotrimoxazole		

Mali	Chloroquine	14	6 - 60 months
			
	Amodiaquine	28	
			
	Sulphadoxine-pyrimethamine		

Sudan	Chloroquine	28	6 months - 7 years
			
	Sulphadoxine-pyrimethamine		
			
	Artesunate + sulphadoxine-pyrimethamine		

Fingerprick blood samples were collected onto filter paper from each individual at the time of recruitment to the study, for genotyping of the parasites present and for characterisation of the human SNP markers used in the study.

### Ethical considerations

The study protocol was reviewed and approved by the Institutional Review Boards of the respective participant countries. Individuals were recruited to the study with the consent of their parents or guardians (for children), or with their own consent.

### Definition of *in vivo *drug resistance and sensitivity

The clinical outcomes of treatment were defined according to WHO recommendations [25]. Samples were analysed for markers of parasite drug resistance from those patients who successfully cleared their infection ("sensitive" or "adequate clinical and parasitological response (ACPR)*" *as well as from those meeting the criteria for treatment failure. Briefly, "sensitivity" is defined as the clearance of parasites following drug treatment, without subsequent recrudescence within a defined period (28 days). An adequate clinical and parasitological response (ACPR) is defined as the absence of parasitaemia on day 28 irrespective of axillary temperature, without previously meeting any of the criteria for early and late treatment failure [25].

### Molecular characterisation of drug resistance

Molecular analysis of parasite DNA from patients was performed according to standard IAEA protocols [32,33]. In all studies, parasites appearing during the follow-up period were characterised to distinguish possible reinfections from genuine recrudescence of resistant parasites, according to standard methodology [32].

DNA was extracted from the filter paper samples taken at admission to the study (i.e. before treatment), and amplified with primers to the genes in *P. falciparum *previously reported to be involved in resistance to chloroquine (*Pfcrt, Pfmdr1*) and to SP (*dhfr, dhps)*. The PCR product for each gene was then analysed using dotblot or RFLP to characterize the mutations present that have been linked to resistance [33]. The set of polymorphisms within drug resistance genes which were used to define drug resistance was defined for each country based on previous studies (Table 2).

**Table 2:** Molecular definition of drug-resistance according to participant countries

Country	Definition of genotypic resistance to:		
	
	Chloroquine	SP	Other
Burkina Faso	*Pfcrt76*T	*Dhfr51*I*/59*R*/108*N	

Cameroon (Yaoundé)	n/a	*Dhfr51*I*/59*R*/108*N *+ Dhps437*G	AQ: *Pfcrt76*T + *Pfmdr1-86*Y

Kenya	*Pfcrt76*T	*Dhfr108*N + one or more of *Dhfr51*I, *Dhfr59*R, *Dhps436*A, *Dhps540*E	

Mali	*Pfcrt76*T	*Dhfr51*I*/59*R*/108*N	

Sudan	*Pfcrt76*T + *Pfmdr1-86*Y	*Dhfr51*I*/108*N *+ Dhps437*G/*540*E	

Mutations in *Pfcrt *and *Pfmdr1 *were considered for resistance to chloroquine, and in *dhfr *and *dhps *for resistance to SP. SP = sulfadoxine-pyrimethamine AQ = amodiaquine. n/a = not applicable.

Cases of mixed infection, i.e. infections with both the wild-type and the resistance (mutant) allele, were considered as resistant. Only those samples that carried resistant alleles were included in the analysis and were divided into two groups (i) the cases: drug-resistant parasite genotype but infection was cleared following drug treatment, and (ii) the controls: drug-resistant parasite genotype and infection not cleared following treatment.

### SNP genotyping of human DNA

Human DNA was extracted from filter paper blood samples (1 ml), drawn at the time of enrolment, using the Nucleon BACC2 DNA extraction Kit (Amersham Pharmacia Biotech, Buckinghamshire, UK), according to the manufacturer's protocol. The concentration of DNA was determined using the PicoGreen^® ^double strand (dsDNA) DNA Quantification Kit (Molecular Probes, Inc.). In order to increase the amount of human DNA required for high-throughput genotyping, all samples were subjected to whole genome amplification by primer extension pre-amplification PCR, using 15N base primers http://www.genetix.com[34]. The thermal cycling parameters were: 1 cycle at 94°C for 3 minutes for an initial denaturation, followed by 50 cycles of denaturation for 1 min at 94°C, primer annealing for 2 min at 37°C, 0.1°C/sec to 55°C, primer extension for 4 min at 55°C; and a final extension for 5 minutes at 72°C as described [35]. Amplified DNA samples were used at 1:10 dilution for genotyping on the SEQUENOM^® ^iPLEX^® ^platform according to the manufacturer's instructions.

### Selection of human immune response gene variants and genotyping

Known candidate gene variants were selected from the growing list of cytokines and other immune mediators that are thought to be involved in malarial pathogenesis, together with their receptors and promoters. In addition, lymphokines that regulate their expression and the adhesion molecules and inflammatory mediators that mediate their pathological effects were included. SNPs were selected using information from the literature and dbSNP [36], and reflected a compromise between SNP function, marker spacing and minor allele frequency (MAF). The initial SNP selection consisted of validated markers with minor allele frequency (MAF) ≥ 5%. This was narrowed down to an economic 67 known SNPs (Table 3) for which genotyping assays could be designed into two multiplex reactions for the Sequenom^® ^iPLEX^® ^mass spectrometry platform http://www.sequenom.com[37-39]. Genotyping accuracy was assessed by testing the conformation of the observed genotype distributions in the controls to the expected distributions under Hardy-Weinberg equilibrium (HWE). Assays which deviated from HWE at the 0.1% significance threshold were excluded from further analysis.

**Table 3:** Polymorphisms genotyped using SEQUENOM^® ^iPLEX^®^

Alternative Name*	rsnumber	gene	chr	coord	ancestral/reference allele^‡^	derived allele
	rs1803632	GBP7	1	89582690	G	C

Duffy - FyA/FyB	rs2814778	DARC	1	159174683	T	C

	rs2179652	RGS2	1	192769826		

	rs3024500	IL10	1	206940831	G	A
IL10-1082	rs1800896	IL10	1	206946897	T	C
IL10-3533	rs1800890	IL10	1	206949365	A	T

McC (McCoy)	rs17047660	CR1	1	207782856	A	G

SI (Swain-Lagley)	rs17047661	CR1	1	207782889	A	G

IL1A G4845T	rs17561	IL1A	2	113537223	C	A

IL1B A2	rs1143634	IL1B	2	113590390	G	A

	rs708567	IL17RE	3	9960070	C	T

	rs352140	TLR9	3	52231737		
	rs187084	TLR9	3	52261031	G	A

	rs6780995	IL17RD	3	57138419	G	A

	rs4833095	TLR1	4	38799710	C	T
	rs5743611	TLR1	4	38800214	C	G

	rs5743810	TLR6	4	38830350	G	T
	rs5743809	TLR6	4	38830514	A	G

	rs1801033	C6	5	41199959	T	G

	rs2706384	IRF1	5	131826880	G	T

	rs20541	IL13	5	131995964	G	A

IL-4-589	rs2243250	IL4	5	132009154	C	T

LTA +77	rs2239704	LTA	6	31540141	C	A
LTA NCO1	rs909253	LTA	6	31540313	A	G

TNFa -1031	rs1799964	TNF	6	31542308	T	C
TNF -376	rs1800750	TNF	6	31542963	G	A
TNF -308	rs1800629	TNF	6	31543031	G	A
TNF -238	rs361525	TNF	6	31543101	G	A
TNF +851	rs3093662	TNF	6	31544189	A	G

	rs2242665	CTL4	6	31839309	C	T

	rs1555498	IL20RA	6	137325847	C	T

	rs2075820	NOD1	7	30492237	C	T

CD36 T1264G	rs3211938	CD36	7	80300449	T	G

CD36 G1439C	None assigned	CD36	7	80302110	G	C

	rs17140229	CFTR	7	117230283	T	C

	rs4986790	TLR4	9	120475302	A	G

	rs4986791	TLR4	9	120475602	C	T

	rs8176746	ABO	9	136131322	G	T

HbE	rs33950507	HBB	11	5248173	C	T

HbS	rs334	HBB	11	5248232	T	A

	rs7935564	TRIM5	11	5718517	G	A

	rs542998	RTN3	11	63487386	T	C

	rs2227507	IL22	12	68642647	T	C
	rs1012356	IL22	12	68644618	A	T
	rs2227491	IL22	12	68646521	T	C
	rs2227485	IL22	12	68647713	G	A
	rs2227478	IL22	12	68648622	G	A

	rs229587	SPTB	14	65263300	T	C

	rs2230739	ADCY9	16	4033436	T	C
	rs10775349	ADCY9	16	4079823	C	G

	rs1805015	IL4R	16	27374180	T	C

	rs2535611	ADORA2B	17	15861332	C	T

	rs2297518	NOS2	17	26096597	G	C
NOS2A -954 (or -969)	rs1800482	NOS2	17	26128509	C	G
NOS2A -1173	rs9282799	NOS2	17	26128728	G	A
NOS2A -1659	rs8078340	NOS2	17	26129212	G	A

	rs373533	EMR1	19	6919624	C	A

	rs461645	EMR1	19	6919753	A	G

ICAM1 codon241	rs1799969	ICAM1	19	10394792	G	A

ICAM1 codon469	rs5498	ICAM1	19	10395683	A	G

	rs2057291	GNAS	20	57472043		
	rs8386	GNAS	20	57485812	C	T

	rs1128127	DERL3	22	24179132	G	A

Amelogening_SNP1	None assigned	AMELX	X	11313735	G**	A***
Amelogening_SNP2	None assigned	AMELX	X	11316106	T**	C***
Amelogening_SNP6	None assigned	AMELX	X	11316650	C**	A***

CD40LG -727	rs3092945	CD40LG	X	135729609	T	C
CD40LG +220	rs1126535	CD40LG	X	135730555	T	C

G6PD +376	rs1050829	G6PD	X	154110298	T	C
G6PD +202	rs1050828	G6PD	X	154111023	C	T

All SNPs are referenced to dbSNP130 and Ensembl build 56.*Alternative name from the literature or from laboratory usage. ^‡^Ancestral alleles are taken from dbSNP130 and where not identified a reference allele is given based on the human reference sequence on Ensembl. All alleles are with respect to the positive strand. ** Allele represented on the × chromosome and *** Allele represented on the Y chromosome.

### Statistical analyses

Comparisons of age and gender of participants, and parasitaemia at recruitment, for each country, and for all countries pooled, between those who did and did not clear genotypically resistant parasites, were compared using chi-squared tests (for frequency data), Kolmogorov-Smirnov (K-S) tests (for non-normally distributed values) or ANOVA (for normally distributed values). Each SNP was tested for association with the clearance phenotype using Odds Ratio (Univariate allele-based association tests). The data were then adjusted for confounding factors of age, ethnicity, gender and study location.

The P-values were not corrected for multiple testing. The Bonferroni correction, a commonly used correction which assumes independence between markers, was considered too stringent in this study as several SNPs may exhibit high degrees of dependence with one another, as measured by LD (D') [40].

The large overall sample size resulting from combining studies at different sites increases the power to detect true positive associations and reject false-positives. Inter-study heterogeneity in association was assessed using Cochran's chi-square test (Q-test) under the null hypothesis of homogeneity (significant heterogeneity *P < 0.05*). Individual SNPs were investigated using allele- and genotype-based models.

## Results

### Patient samples

A total of 3,721 samples from five countries (Table 4) were found to contain "genotypically resistant" parasites according to the criteria in Table 2. Of these patients, 2,057 (55%) were able to successfully clear their infection. With the exception of Kenya (47.7% cleared resistant infections), more than 50% of individuals in the study were able to clear genotypically resistant parasites (47.7%-76.4%).

**Table 4:** Characteristics of patients with genotypically resistant parasites

Country	Burkina Faso			Cameroon			Kenya			Mali			Sudan			Total		
	**Cleared**	**Not cleared**	**Total**	**Cleared**	**Not cleared**	**Total**	**Cleared**	**Not cleared**	**Total**	**Cleared**	**Not cleared**	**Total**	**Cleared**	**Not cleared**	**Total**	**Cleared**	**Not cleared**	**Total**

Number of samples	264	235	499	730	517	1247	656	718	1374	115	104	219	292	90	382	2057	1664	3721

Median age in years	5.5	7.3	-	4	6	-	11	12	-	3	3	-	14	10		5	5.3	-

Number male gender	37	186	223	371	257	628	265	317	582	78	38	116	123	66	189	874	867	1741

Number female gender	227	49	276	359	260	619	391	401	792	37	66	103	169	24	193	1183	797	1980

Parasitaemia: median parasite density (parasites per μl)	19 960	21 070	-	27015	22075	-	22 160	21360	-	19 630	17215	-	23 360	24380	-	22 425	21 220	-

Parasitaemia: range (parasites per μl)	25 - 44 870	25 - 38 990	-	25 - 26 870	25 - 31 990	-	25 - 23 870	25 - 48 190	-	75 - 179 870	75 - 188 310	-	25 - 27 870	25 - 28 090	-	25 - 25 870	25 - 40 213	-

In contrast to previous studies, there was no significant difference in age overall between those patients who successfully cleared their infection (median 5 years) compared to those who did not (median 5.3 years; K-S test = 47.0; P = 0.16; Table 4). This could be because the data were pooled from five countries with different levels of acquired immunity, and involving different age groups according to the study design chosen. Individuals from highly malaria-endemic areas would be expected to have a higher potential to clear parasites than much older individuals from less endemic areas, so the influence of age is masked by pooling. There was no difference in the gender of patients who successfully cleared their infection and those who did not in Cameroon (χ^2 ^test, P = 0.69) and Kenya (χ^2 ^test, P = 0.16). However in Burkina Faso and Sudan, significantly fewer males and more females than expected successfully cleared a drug resistant infection (χ^2 ^tests: BF: P = 2.5 × 10^-48^, RR = 0.2; Sudan P = 2.2 × 10^-7^, RR = 0.74). By contrast, in Mali significantly more males and fewer females than expected were able to cure a drug resistant infection (χ^2 ^test, P = 3.6 × 10^-6^, RR = 1.87). These apparent gender effects could however be the result of significant differences in the age of male and female participants in some countries in the study. In Burkina Faso and Sudan, the median age of females was significantly higher than that of males (K-S test, P = 0.04 (BF); P = 0.02 (Sudan), whereas in Mali, male participants were older, although this did not quite reach statistical significance (K-S test, P = 0.06). Previous studies suggest that overall, older children are more likely to clear drug-resistant infections than younger children [10]. The parasitaemia at admission to the study in those who cleared and did not clear their infections was not significantly different for any of the five countries (K-S tests: Burkina Faso (P = 0.18), Cameroon (P = 0.84), Kenya (P = 0.65), Mali (P = 0.37), and Sudan (P = 0.29)).

### Single-SNP analysis

Cochran's chi-test of heterogeneity revealed sufficient homogeneity between the studies at the loci discussed for the application of meta-analysis (P < 0.05). An initial analysis of association of each of the 70 SNPs with clearance of drug resistant parasites revealed 17 SNPs those were significantly associated with the phenotype (Table 5 P ≤ 0.05). Three SNPs (rs1799969, rs1126535 and rs2814778) showed a strong association with the clearance phenotype with p-values less than 10^-5 ^(Table 5).

**Table 5:** Univariate allele-based association tests

SNP*	Allele 1/2	Clearance		Non-clearance		Chi-squared	p-value
		
		Allele 1	Allele 2	Allele 1	Allele 2		
rs1012356	A/T	0.53	0.47	0.49	0.51	11.38	0.007

rs2227491	C/T	0.57	0.43	0.60	0.40	7.02	0.008

rs2227485	A/G	0.44	0.56	0.47	0.53	10.9	0.0009

rs2227478	A/G	0.64	0.36	0.66	0.34	5.89	0.02

rs2706384	A/C	0.41	0.59	0.46	0.54	18.9	0.00001

rs2057291	A/G	0.19	0.81	0.19	0.81	0.17	0.68

CD36 G1439C	C/G	0.01	0.99	0.01	0.99	1.49	0.22

**rs1799969**	**A/G**	**0.04**	**0.96**	**0.06**	**0.94**	**15.83**	**7.10^-6^**

rs20541	C/T	0.76	0.24	0.74	0.26	6.86	0.009

rs1800750	A/G	0.05	0.95	0.05	0.95	0.70	0.40

rs3024500	A/G	0.62	0.38	0.63	0.37	2.10	0.15

rs1805015	C/T	0.37	0.63	0.36	0.64	1.39	0.24

rs17047660	A/G	0.72	0.28	0.74	0.26	8.13	0.004

rs17047661	A/G	0.42	0.58	0.45	0.55	7.20	0.007

rs1714022	C/T	0.29	0.71	0.27	0.73	2.80	0.09

**rs1126535**	**C/T**	**0.22**	**0.78**	**0.28**	**0.72**	**31.13**	**10^-6^**

rs2230739	A/G	0.86	0.14	0.86	0.14	0.54	0.46

rs229587	C/T	0.39	0.61	0.42	0.58	4.37	0.04

**rs2814778**	**A/G**	**0.14**	**0.86**	**0.20**	**0.80**	**55.17**	**10^-6^**

rs3092945	C/T	0.32	0.68	0.29	0.71	10.69	0.001

rs1128127	A/G	0.44	0.56	0.47	0.53	8.19	0.004

rs1803632	C/G	0.51	0.49	0.53	0.47	4.84	0.03

rs7935564	A/G	0.46	0.54	0.45	0.55	2.39	0.12

rs4833095	C/T	0.84	0.16	0.82	0.18	5.23	0.02

rs5743809	C/T	0.06	0.94	0.05	0.95	4.92	0.03

For each SNP, chi-squared comparisons were made of between the allele frequencies found in patients who cleared and did not clear drug resistant parasites. Highly significant associations are highlighted in bold. *Of the 67 SNPs genotyped for association, 42 SNPs were not included in the analysis because they were either monomorphic in one or more countries (n = 19) or for deviation from HWE in one or more of the participant countries (n = 23).

Further analysis using genotype-based tests (Table 6) indicated a highly significant association (P < 0.01) of 9 SNPS with the clearance of resistant parasites. 19 of the 25 SNPs showed a significant association (P < 0.05) with clearance (Table 6). After adjusting the data for age, ethnicity, gender and study location, three SNPs remained significantly associated with the clearance phenotype: SNP rs2706384 (OR = 0.76 [95%, CI: 0.64 - 0.92]; p = 0.005), SNP rs1128127 (OR = 0.77 [95%, CI: 0.59 - 0.99], p = 0.05), and SNP rs2057291 (OR = 1.27 [95%, CI: 1.02 - 1.57], p = 0.03). No other SNPs were statistically associated with the clearance phenotype (Table 6).

**Table 6:** Genotype association analysis

SNP (genotype model)	Genotype-based tests*		Adjusted analysis**		Multiple SNP analysis***	
	
	Chi-squared	p-value	OR [95%, CI]	p-value	OR [95%, CI]	p-value
rs1012356 (TT vs AT/AA)	**17.20**	**0.001**	1.15 [0.90 - 1.46]	0.27	1.23 [0.95 - 1.85]	0.09

rs2227491 (TT vs CT/CC)	6.90	0.07	1.04 [0.80 - 1.34]	0.78	0.97 [0.68 - 1.39]	0.88

rs2227485 (AA vs GG/AG)	**11.30**	0.01	1.04 [0.82 - 1.32]	0.74	0.89 [0.64 - 1.25]	0.50

rs2227478 (GG vs AA/AG)	6.60	0.09	1.14 [0.86 - 1.51]	0.36	1.21 [0.82 - 1.80]	0.34

**rs2706384** **(AA vs CC/AC)**	**21.80**	**0.0001**	**0.76** **[0.64 - 0.92]**	**0.005**	**0.76** **[0.71 - 0.92]**	**0.005**

rs2057291 (AA vs GG/AG)	11.90	0.02	1.27 [1.02 - 1.57]	0.03	0.91 [0.71 - 1.17]	0.47

CD36 G1439C (CC vs GG/CG)	9.30	0.03	-	-	-	-

ICAM1 CODON241 (AA vs GG/AG)	**30.03**	**0.0001**	0.98 [0.71 - 1.36]	0.92	1.04 [0.74 - 1.44]	0.84

rs20541 (TT vs CC/CT)	**23.90**	**0.0001**	0.89 [0.67 - 1.19]	0.44	1.07 [0.75 - 1.54]	0.70

TNF -376 (AA vs GG/AG)	**13.40**	**0.004**	1.17 [0.69 - 1.98]	0.56	0.95 [0.50 - 1.80]	0.87

rs3024500 (GG vs AA/AG)	8.80	0.03	1.04 [0.77 - 1.41]	0.79	1.10 [0.78 - 1.58]	0.60

**RS1805015** **(CC vs TT/CT)**	8.70	0.03	0.84 [0.65 - 1.10]	0.21	0.66 [0.45 - 0.97]	0.03

rs17047660 (GG vs AA/AG)	7.20	0.07	0.95 [0.72 - 1.23]	0.68	1.32 [0.84 - 2.07]	0.23

rs17047661 (AA vs GG/AG)	7.40	0.06	0.88 [0.66 - 1.16]	0.36	0.72 [0.49 - 1.05]	0.09

rs17140229 (CC vs TT/CT)	8.60	0.04	1.08 [0.79 - 1.46]	0.62	1.41 [0.94 - 2.10]	0.09

rs1126535 (CC vs TT/CT)	**21.60**	**0.0001**	0.91 [0.67 - 1.22]	0.52	0.85 [0.51 - 1.40]	0.51

rs2230739 (GG vs AA/AG)	10.40	0.02	1.06 [0.80 - 1.39]	0.69	1.26 [0.82 - 1.92]	0.29

rs229587 (CC vs TT/CT)	8.50	0.04	0.97 [0.76 - 1.22]	0.76	1.02 [0.73 - 1.43]	0.92

rs2814778 (AA vs GG/AG)	**33.03**	**0.0001**	0.82 [0.53 - 1.28]	0.38	0.85 [0.53 - 1.36]	0.50

rs3092945 (CC vs TT/CT)	**10.60**	0.01	0.83 [0.61 - 1.11]	0.21	0.89 [0.60 - 1.34]	0.60

**rs1128127** **(AA vs GG/AG)**	**12.04**	**0.007**	0.77 [0.59 - 0.99]	0.05	0.67 [0.45 - 0.99]	0.05

rs1803632 (GG vs CC/CG)	7.04	0.071	0.78 [0.58 - 1.05]	0.10	0.76 [0.49 - 1.17]	0.21

rs7935564 (AA vs GG/AG)	**21.10**	**0.0001**	1.02 [0.84 - 1.25]	0.81	0.93 [0.70 - 1.22]	0.59

rs4833095 (TT vs CC/CT)	6.90	0.08	0.75 [0.52 - 1.07]	0.12	0.86 [0.57 - 1.33]	0.50

rs5743809 (CC vs TT/CT)	11.40	0.01	1.25 [0.57 - 2.74]	0.59	1.23 [0.53 - 2.84]	0.63

Highly significant associations (P < 0.01) are highlighted in bold; significant associations (0.01 < P < 0.05) are underlined. OR = odds ratio; *Single locus based tests; **Each SNP adjusted for age, ethnic group, gender, and study location. ***Multiple SNPs adjusted for ethnic group, age, gender, and study location.

### Multiple SNP analysis

A multiple SNP analysis was performed in order to correct for covariate effects (multiple SNPs adjusted for ethnic group, age, site, and gender) (Table 6). The main predictive factor of clearance was age as has been reported previously by others [10,41-43]. Two of the three SNPs identified in the single SNP analysis remained significantly associated with the clearance phenotype: SNP rs2706384 (OR = 0.76 [95%, CI: 0.71 - 0.92]; p = 0.005) and SNP rs1128127 (OR = 0.67 [95%, CI: 0.45 - 0.99], p = 0.05). One additional SNP (rs1805015) was now found to be associated with the clearance phenotype (OR = 0.66 [95% CI: 0.45 - 0.97], p = 0.03).

## Discussion

The malaria parasite has had a substantial evolutionary influence upon the genetic constitution of its human host (recently reviewed in [44]). Individuals living in malaria-endemic regions seem to develop an ability to clear drug-resistant parasites (following treatment) as they get older [10], which is presumably the result of increasing acquired immunity. The influence of human host polymorphisms in immune response-type genes on the likelihood of clearance of drug-resistant parasites has so far received little attention.

In the present study, a single SNP locus analysis was carried out to investigate the contribution of the host genetic factors in the clearance of drug-resistant parasites following treatment, by examining the classic, previously published SNPs, which may play a critical role in individuals' ability to clear drug-resistant malaria parasites. The effects of polymorphisms in a number of genes, including β-globin, G6PD, TNF-α, IFN-γ, CD36, ICAM-1, IL10, IL4R, and LTA (Table 3), upon the clearance of malaria parasites in African individuals was investigated across five large association studies from Burkina Faso, Cameroon, Kenya, Mali, and Sudan.

Amongst the 70 SNPs investigated in this study, seventeen were found at significantly different frequencies (P < 0.05) in people who cleared drug resistant infections than those who did not (Table 5). Further analysis using genotype-based tests indicated that nine SNPs were strongly associated (P < 0.01) with parasite clearance (Table 6). Following adjustments for the possible confounding factors of age, ethnicity, gender and study location, and analysing multiple SNPS to correct for covariate effects, three SNPs remained significantly associated with the clearance phenotype, across Africa and with three different drugs. It is, however, important to note that the demonstration of association with clearance phenotype of these three SNPs does not necessarily imply that any of the SNPs are functional in the clearance of drug-resistant parasites. The SNPs may simply be reflecting the signal of a different functional variant(s) in moderate-to-high LD with them [45].

**SNP rs2706384 **is in the 5' upstream region of the interferon regulatory factor *IRF1 *gene, 1710 bp upstream of the ATG start codon and -415bp from the transcriptional start site. Individuals with a homozygous AA genotype at this locus were significantly less likely to clear drug resistant infections than those homozygous CC or heterozygous (OR = 0.76 [95% CI: 0.71 - 0.92]; P = 0.005). The A allele was found more frequently in individuals who did not clear their drug-resistant infection than in those who did (P = 0.00001).

IRF-1 is a transcription factor that has been shown to regulate expression of a number of genes involved in both innate and adaptive immunity, notably TLR9, MHC Class I and II genes, IL-15, iNOS in macrophages, IL-4 and IL-12/p40 [46]. Interferon-γ, the strongest inducer of IRF-1, is thought to be a key player in the control of pre-erythrocytic and blood stage infection, both in rodent malaria infections [47] and in human malaria infections [48,49]. Healthy individuals homozygous AA at rs2706384 were found to have significantly higher *IRF-1 *mRNA expression than CC homozygotes [50]. Thus individuals of the AA genotype may produce higher levels of IRF-1 in response to the same IFN- γ stimulus, which may shift the balance more towards a Th1 response and away from a Th2 response through repression of IL-4 transcription and increased IL-12/p40 expression. This suggests that antibody may play a key role in the control of drug-resistant parasites. In addition, the binding of NF-kappa B to the C allele was significantly higher than to the A allele [50]; this transcription factor may have a negative regulatory role in IFN-induced gene expression [51,52].

Previous work has shown an association of the same SNP, rs2706384, with protection against *P. falciparum *infection in two West African ethnic groups [53]. However, in that study the C allele was associated with a higher risk of having a *P. falciparum *infection for Mossi but not for Fulani, and in Fulani CC and AA individuals were more frequently parasitized than heterozygous individuals.

**Rs1805015 **is a missense mutation (Ser503Pro) within the insulin-IL4 receptor motif (I4R) of the alpha subunit of the interleukin4 receptor gene *IL4R*. Individuals with a homozygous CC genotype (encoding 503Pro) at this locus were significantly less likely to clear drug resistant infections than those who were homozygous TT or heterozygous (OR = 0.66 [95%, CI: 0.45 - 0.97], p = 0.03), but there was no significant difference in the frequency of the C allele in individuals who cleared or did not clear their drug-resistant infection (P = 0.24), suggesting that the failure to clear infections was associated with the CC homozygote.

The interaction of IL-4 with its receptor results in binding of JAK to the I4R motif of IL4R-α; however, this binding is unaffected by the Ser503Pro substitution [54]. The Ser503Pro substitution appears to reduce the subsequent binding and phosphorylation of STAT6 [54]. Since phosphorylated STAT6 controls cell differentiation and gene transcription [55], the Ser503Pro substitution could therefore lead to a reduction in response to IL-4, such as reduced B-cell proliferation and antibody production, further supporting the role of Th2 responses in the clearance of drug-resistant parasites.

However, a previous malaria case-control study in Sudan found the CC genotype to be at a significantly lower frequency in malaria cases compared to non-malaria controls in Sudan [56], whereas in this study CC genotypes were less able to clear drug resistant infections than individuals of genotype AA or AC. Individuals with the Ser503Pro *IL4R *mutation were found to have lower IgE levels [54] and a separate study found a significant association with atopy and asthma-related phenotypes [57].

**SNP rs1128127 **is a missense mutation (Ala211Val) within the Der1-like domain family gene *Derl3*. Individuals with a homozygous AA genotype (encoding 211Val) at this locus were significantly less likely to clear drug resistant infections than those who were homozygous GG or heterozygous (OR = 0.77 [95%, CI: 0.45 - 0.99], P = 0.05). The A allele was also found more frequently in individuals who did not clear their drug-resistant infection than in those who did (P = 0.004). This suggests that GG or AG individuals have an advantage over AA genotypes in their ability to control drug-resistant infections.

The derlin family of proteins are found in the endoplasmic reticulum (ER) and are thought to be involved in the degradation of misfolded glycoproteins within the ER [58-61]. *Derl3 *is expressed at high levels in specific tissues such as the placenta, pancreas, small intestine and spleen, whereas other members of the family have more widespread expression [61]. There does not appear to be any previous study linking mutations in *Derl3 *to the control of infectious disease. The frequency of heterozygous AG individuals is much higher in sub-Saharan Africans (0.65) than in Europeans (0.183) [62], which could be explained by positive selection of heterozygous AG individuals in populations exposed to malaria, because of their enhanced ability to clear (drug- resistant) parasites.

Host genetic factors such as cytokines may be the key determinants of malaria severity and outcome. Several studies suggest that the balance between pro- (TNF-α, IFN-γ, IL-8) and anti-inflammatory (IL4, IL-10, TGF-β) cytokines determines the degree of malaria parasitaemia, the level of anaemia, the clinical severity, the presentation, and/or the outcome of infection [63-65]. IFN-γ has been suggested to be a key molecule in human anti-parasite host defence, and appears to be essential for the control of parasitaemia. The role of IL4 is less clear; some studies have not supported direct involvement of IL-4 (or IL-13) in the clearance of *P. falciparum *parasites [63], and IL-4 has been shown to suppress macrophage-mediated killing of *P. falciparum in vitro *[66].

This study is the first to assess the role of specific human genetic variants (SNPs) in the clearance of drug-resistant parasites after anti-malarial treatment. Three SNPs were found to be strong predictors of the clearance of drug-resistant parasites, even after correction for age, ethnicity, gender and study location. Two of the three SNPs identified are in loci associated with pro-inflammatory (interferon- γ) and anti-inflammatory (IL-4) cytokine responses.

The assessment of the role of human genetic determinants may improve understanding of the interface between host immunity and anti-malarial drug resistance. The relationship between host polymorphisms and malaria parasite clearance is complex, and larger studies in other settings will be required, both to confirm these associations, to investigate further the weak associations, and also to investigate the contribution of the host immunological factors and the parasite *per se *in the clearance of drug-resistant parasites.

## Conclusions

The study has identified a significant association of three loci in the human genome with the ability of parasite to clear drug-resistant *P. falciparum*. One locus, a SNP in the promoter region of the *IRF-1 *gene, has previously been linked to the control of malaria parasite density, and it is possible that patients able to clear drug-resistant infections have an enhanced ability to control parasite growth, perhaps through a more Th2-biased T cell response. The association of clearance with a SNP within the IL-4R gene, that possibly reduces the response to IL-4, supports the hypothesis that a stronger Th2 response assists clearance of drug-resistant parasites. The third locus encodes a protein involved in the degradation of misfolded proteins within the endoplasmic reticulum, and its role, if any, in the clearance phenotype needs to be further investigated.

## Competing interests

The authors declare that they have no competing interests.

## Authors' contributions

Data were collected, and molecular genotyping for drug resistance markers performed, by individual teams from Burkina Faso (JBO, IZ), Cameroon (WFM, OA, MSBE, EAA), Kenya (FK, SOJ), Mali (MMK, AD) and Sudan (NT, IM). Human polymorphisms were genotyped by MD, AG, CH, AJ, KR, KR, DPK. LRC supervised molecular drug resistance genotyping and provided positive controls and SOPs. MD provided SOPs for human polymorphisms genotyping and data analysis. MD, LRC, DPK, BK, KR conceived of the study and participated in its design and coordination. MD and LRC drafted the manuscript, with additional comments and input from DPK, BK and KR. All authors read and approved the final manuscript.
